# Comparison of Morphological Characteristics, Histological Tissue Structures, and Intestinal Function Among Eight Ornamental Fish Species Under Identical Aquaculture Conditions

**DOI:** 10.3390/biology15131043

**Published:** 2026-06-30

**Authors:** Mingxin Xie, Bing Fu, Jiun-Yan Loh, Ning Yang, Minyi Zhong, Pan Chen, Chaojie Yang, Hai Huang, Bing Chen, Yan Chen

**Affiliations:** 1 Yazhou Bay Innovation Institute, Hainan Tropical Ocean University, Sanya 572024, China; xiemingxin5@126.com (M.X.); dayangning@163.com (N.Y.); cpanpan@163.com (P.C.); yangchaojie1986@163.com (C.Y.); huanghai74@126.com (H.H.); 2 Hainan Key Laboratory for Conservation and Utilization of Tropical Marine Fishery Resources, Sanya 572000, China; 3 Key Laboratory of Utilization and Conservation for Tropical Marine Bioresources, Ministry of Education, Hainan Tropical Ocean University, Sanya 572022, China; 4 Institute of Animal Science, Guangdong Academy of Agricultural Sciences/Aquatic Research Center, Guangdong Academy of Agricultural Sciences/Key Laboratory of Animal Nutrition and Feed Science in South China, Ministry of Agriculture and Rural Affairs/Guangdong Key Laboratory of Animal Breeding and Nutrition, Guangzhou 510640, China; 15869704640@163.com (B.F.); minyizhong2022@126.com (M.Z.); 5 Tropical Futures Institute, James Cook University (Singapore Campus), 149 Sims Drive, Singapore 387380, Singapore; james.loh@jcu.edu.au

**Keywords:** ornamental fish, intestinal microbiota, morphological structure, health, function

## Abstract

The intestine, particularly the gut microbiota, and the liver play key roles in digestion, nutrient transformation, and immune regulation in fish. However, limited information is available regarding how different ornamental fish species regulate these systems under identical aquaculture conditions. Therefore, this study systematically compared gut microbiota diversity, structural variation, and predicted ecological functions among eight ornamental fish species reared in the same environment, using 16S rRNA high-throughput sequencing combined with digestive enzyme indices and histological analysis of intestinal and liver tissues. The results showed that goldfish (*Carassius auratus*) and crucian carp exhibited efficient digestive and absorptive capacities, supported by a thickened muscularis and prominent mucosal layers (*p* < 0.001). High goblet cell density was observed in red swordtail (*Xiphophorus hellerii*) and Mickey Mouse platy (*Xiphophorus hellerii × X. maculatus*) (*p* < 0.001). Larger hepatocyte perimeter and area were observed in red swordtail (*p* = 0.022, *p* = 0.015), whereas platinum mini parrot cichlid and sapphire mini parrot cichlid showed significant hepatocyte vacuolization. Microbial analysis showed that the eight fish species had similar α diversity indices, while the gut microbial profiles of Mickey Mouse platy and golden crucian carp differed the most. At the genus level, beneficial taxa such as *Lactococcus, Paracoccus*, and *Cetobacterium* were significantly enriched in red swordtail, sailfin molly, and goldfish, respectively, whereas opportunistic pathogens, including *Edwardsiella, Aeromonas*, and *Acinetobacter*, were enriched in Mickey Mouse platy, sapphire mini parrot cichlid, and golden crucian carp, respectively (*p* < 0.05). Functional prediction based on KEGG pathways indicated that sailfin molly and Mickey Mouse platy exhibited the broadest functional enrichment, primarily involving amino acid metabolism, fatty acid metabolism, and antibiotic biosynthesis. Crucian carp and golden crucian carp showed higher activity in amino acid biosynthesis and glycolysis/gluconeogenesis pathways. The two parrot cichlid species were characterized by enrichment in biofilm formation pathways of pathogenic bacteria and amino sugar and nucleotide sugar metabolism pathways. Goldfish and red swordtail were mainly associated with quorum sensing and ABC transporter pathways. These results provide a theoretical foundation for optimizing aquaculture conditions for ornamental fish and improving fish health and production efficiency.

## Simple Summary

Intestinal microbiota closely affects the growth and health of ornamental fish. In this work, we analyzed the intestinal microbial composition, digestive enzymes and intestinal morphology of eight common ornamental fish species. Combined with microbial functional prediction and correlation analysis, we explored the links between gut microbes, host digestion and growth. The results reveal interspecific differences in fish gut characteristics. This study provides a basic reference for the health management and scientific feeding of ornamental fish in aquaculture.

## 1. Introduction

Over the past two decades, extensive studies have investigated the gut microbiota of fish. The application of conventional bacteriological approaches, together with modern molecular biological techniques, has demonstrated that gut microorganisms co-develop with the host and play an important role in immunity, metabolism, growth, and evolution [1,2]. The complexity of the fish gut microbiome is essential for nutrient absorption, intestinal immune function, and physiological regulation [3,4]. The intestinal microbial composition differs among fish species and is affected by multiple external and host-associated factors, including developmental stage, nutritional status, sex, and digestive system complexity, all of which contribute to the establishment and diversity of intestinal microbiota [5,6]. In particular, compared to food fish, ornamental fish exhibit significant differences in the closed nature of the aquaculture system, the source of the microbial environment, the purpose of feed additives, and the focus of health risks. Furthermore, cross-species comparisons among multiple ornamental fish species can not only reveal the co-evolutionary relationship between host phylogeny and microbial communities but also provide direct evidence for polyculture management and the development of species-specific probiotics [7,8].

The global ornamental fish market reached approximately USD 5.88 billion in 2022 and is projected to expand at a compound annual growth rate of 8.5% from 2023 to 2030 [9]. China is one of the world’s major producers of freshwater ornamental fish, and the output value of the ornamental fish industry reached USD 1.65 billion in 2022, representing a year-on-year increase of 16.93% [10]. The intestinal microbial community of ornamental fish is closely associated with species, developmental stage, feeding habit, season, habitat, sex, and growth status and includes protozoa, fungi, yeasts, viruses, and bacteria [1,5,11,12]. Among these microorganisms, bacteria constitute the dominant microbial population in the fish intestine, with approximately 10^7^–10^8^ bacterial cells per gram of gut contents [13]. Colonization of the fish intestinal microbiota begins after hatching and is primarily derived from feed and the surrounding aquatic environment, with aerobic and facultative anaerobic bacteria representing the predominant microbial groups [14]. Compared with mammals, fish exhibit lower phylogenetic diversity in their gut microbiota, which may be due to the harsher and more variable intestinal environment of aquatic organisms [15].

Feeding habit is considered one of the major factors shaping intestinal microbial diversity in fish and is generally characterized by the pattern herbivorous fish > omnivorous fish > carnivorous fish [16]. The dominant bacterial taxa also differ among fish with different feeding strategies. Anaerobic bacteria belonging to Clostridia are commonly enriched in herbivorous fish, whereas Proteobacteria are frequently dominant in carnivorous and omnivorous fish [17]. With the rapid development of aquaculture, nutritional research and feed optimization have become important approaches for promoting fish growth, maintaining health, and improving adaptation to high-density culture conditions [12].

The fish gut microbiota not only reflects host physiological status but also functions as a dynamic ecological system regulated by multiple factors. Recent studies have highlighted the importance of gut microbiota in fish nutrition and health [18]. In particular, intestinal microorganisms participate in nutrient metabolism and may affect the host’s ability to utilize dietary components efficiently [5]. Furthermore, interactions between intestinal microbiota and the host immune system are essential for maintaining intestinal homeostasis and protecting against pathogen invasion [19]. The distinct microbial composition observed in fish, compared with that of mammals, reptiles, and birds, further demonstrates the evolutionary and ecological specificity of fish-associated microbiota [20]. Moreover, the involvement of gut microorganisms in lipid, carbohydrate, and protein metabolism has attracted increasing attention due to its implications for fish health and aquaculture production [18].

The relationship between intestinal morphology and gut microbiota is bidirectional and functionally important. Intestinal mucosal characteristics, including villus height, crypt depth, and epithelial integrity, determine the available surface area for microbial adhesion and colonization [21]. In turn, commensal microorganisms contribute to intestinal structural development by promoting epithelial proliferation, maturation of the mucus layer, and development of the immune system [22]. Studies using germ-free fish models have shown that the absence of microbiota results in underdeveloped intestinal villi and impaired digestive function [23]. Therefore, the combined investigation of intestinal histological characteristics and microbial communities is essential for understanding host–microbe interactions associated with the health and disease resistance of ornamental fish.

Therefore, eight ornamental fish species examined in this study were selected to represent a broad range of phylogenetic backgrounds, feeding habits, economic significance, and production systems within the ornamental aquaculture industry. Specifically, the selection includes three cyprinids (goldfish *Carassius auratus*, golden crucian carp *Carassius auratus red* var. ♂ × *Cyprinus carpio* L. mirror ♀, and crucian carp *Carassius auratus*), three poeciliids (sailfin molly *Poecilia latipinna*, red swordtail *Xiphophorus hellerii*, and Mickey Mouse platy *Xiphophorus hellerii × X. maculatus*), and two cichlids (platinum and sapphire mini parrot cichlids, *Amphilophus* spp.). This taxonomic coverage (Cyprinidae, Poeciliidae, Cichlidae) allows assessment of whether observed responses to identical aquaculture conditions are consistent across evolutionarily distant lineages [24]. Moreover, the species span diverse feeding strategies—from herbivorous tendency (e.g., sailfin molly) to omnivorous (cyprinids and cichlids) and insectivorous/omnivorous (red swordtail)—enabling comparisons linked to trophic habits [25]. Economically, the chosen species represent major segments of the global ornamental fish trade: goldfish and crucian carp as traditional coldwater aquarium staples; poeciliids as the most widely kept livebearers in tropical freshwater systems; and the hybrid golden crucian carp and mini parrot cichlids as emerging examples of artificially selected or cross-bred ornamental varieties with increasing market share [26]. Thus, the inclusion of these eight species ensures that the findings under identical aquaculture conditions are both phylogenetically informed and directly relevant to different sectors of ornamental aquaculture.

This study systematically compared intestinal and hepatic histomorphological characteristics, together with the diversity and compositional variation of gut microbial communities, among eight ornamental fish species reared under identical aquaculture conditions. High-throughput 16S rRNA gene sequencing combined with histomorphological analysis was employed to investigate microbial community structure and potential ecological functions. The aim was to provide a theoretical basis for optimizing ornamental fish culture feed, improving fish health, and increasing aquaculture efficiency.

## 2. Materials and Methods

### 2.1. Diet and Management

The experiment was conducted at Sanya Aquarium under identical culture conditions for 56 days. A total of 240 ornamental fish belonging to eight species (Supplementary Figure S1) were randomly assigned to 24 culture tanks (35 cm × 45 cm × 35 cm). Each experimental group consisted of three replicates with 10 fish per tank (Table 1). The experimental diet contained 41% crude protein, 5% crude fiber, 16% crude ash, 13% moisture, 4.5% total phosphorus, 2.2% lysine, and 3% crude lipid. All fish were fed the same diet throughout the experiment to ensure consistent nutritional composition and minimize experimental bias associated with dietary differences. Fish were fed daily at 3–5% of body weight at 08:00 and 19:00. Feed intake was recorded daily, and feeding amounts were adjusted according to feeding performance. Daily uneaten feed was siphoned out 30 min post-feeding, dried to constant weight and subtracted from total feed offered to calculate actual feed intake. Continuous aeration was maintained for 24 h to ensure dissolved oxygen levels above 5 mg/L. Water temperature was maintained at 29.8 ± 0.3 °C and pH at 7.8 ± 0.2. Culture water was treated by sedimentation and sand filtration to maintain water quality, and 30–50% of the water was replaced daily to ensure a stable and healthy aquaculture environment. All experimental procedures were carried out following the animal ethics approval (No. 20221111B1) of Hainan Tropical Ocean University.

### 2.2. Sample Collection

At the end of the feeding trial, feed intake was recorded to calculate the feed conversion ratio (FCR) and feeding rate. Five fish were randomly selected from each tank for the measurement of body weight and body length, and weight gain rate, specific growth rate (SGR), and condition factor (CF) were calculated. Under sterile conditions, intestinal contents from five fish per group were collected into 2 mL nuclease-free cryogenic tubes, immediately frozen in liquid nitrogen, and stored at −80 °C for 16S rRNA sequencing analysis. Furthermore, whole intestines from three fish per tank were collected (one composite sample per tank, *n* = 3), stored at −80 °C, and used for digestive enzyme activity assays.

#### 2.2.1. Intestinal Digestive Enzyme Measurement

Whole intestinal tissues (0.1 g) were homogenized in nine volumes (*w*/*v*) of physiological saline and centrifuged at 2500 r/min for 10 min. The resulting supernatant was collected for the determination of digestive enzyme activities according to previously reported methods [27].

#### 2.2.2. Histopathological Analysis of Fish Intestinal and Liver Tissues

Two intestinal and two liver tissue samples were randomly collected from each tank and fixed in 4% formaldehyde for 48 h in 10 mL centrifuge tubes. Tissue samples were subsequently embedded in paraffin, sectioned, and stained with hematoxylin and eosin (H&E). Histological characteristics of hepatocytes, as well as intestinal morphology and structure, were observed under a light microscope (Eclipse Ci-L, Nikon, Tokyo, Japan). Morphometric measurements and image analyses (three times per sample)were performed using Image-Pro Plus 6 software (Media Cybernetics, Rockville, MD, USA) [28].

#### 2.2.3. Intestinal Content Collection and Microbial Community Analysis

At the end of the feeding experiment, intestinal contents from five fish per tank were collected under sterile conditions, transferred into 2 mL nuclease-free cryogenic tubes, immediately frozen in liquid nitrogen, and stored at −80 °C until analysis. Genomic DNA was extracted from intestinal content samples using a PowerSoil DNA Isolation Kit (MoBio, Carlsbad, CA, USA), and DNA concentration and quality were determined according to the manufacturer’s instructions. Approximately 20–30 ng of genomic DNA was used as the template for PCR amplification of the hypervariable V3–V4 regions of the prokaryotic 16S rRNA gene. The forward primer sequence was 5′-CCTACGGRRBGCASCAGKVR VGAAT-3′, and the reverse primer sequence was 5′-GGACTACNVGGGTWTCTAATCC-3′. After amplification, sequencing adaptors and index sequences were added to the PCR products for next-generation sequencing (NGS).

Library concentrations were quantified using a Qubit 3.0 Fluorometer (Invitrogen, Carlsbad, CA, USA). The libraries were normalized to 10 nM and subjected to paired-end 250 bp sequencing on an Illumina NovaSeq 6000 platform (Illumina, San Diego, CA, USA) according to the manufacturer’s instructions. Raw paired-end reads obtained from sequencing were merged by pair-end assembly. Sequences containing ambiguous bases (N) were removed, and only sequences longer than 200 bp were retained. After quality filtering, chimeric sequences were eliminated, and the remaining high-quality sequences were used for operational taxonomic unit (OTU) analysis. Sequence clustering was performed using VSEARCH (version 1.9.6) with a 97% similarity threshold. Taxonomic annotation was conducted against the SILVA 132 16S rRNA reference database for community composition analysis. Representative OTU sequences were classified using the Ribosomal Database Project (RDP) classifier based on the Bayesian algorithm, and microbial community composition was analyzed at different taxonomic levels. Based on OTU analysis, α-diversity indices, including Shannon and Chao1 indices, were calculated using a random subsampling approach, and rarefaction curves were constructed. Unweighted UniFrac analysis was performed to compare microbial community differences among groups. β-diversity was visualized by principal coordinates analysis (PCoA) based on the Bray–Curtis distance matrix. Hierarchical clustering was conducted using the unweighted pair-group method with arithmetic mean (UPGMA). Linear discriminant analysis effect size (LEfSe) analysis was performed to identify microbial biomarkers among groups using the Galaxy platform (http://huttenhower.sph.harvard.edu/galaxy, accessed on 16 April 2023). Redundancy analysis (RDA) was conducted using Canoco software (version 5.0).

Microbial co-occurrence network graphs were generated using Cytoscape version 3.7.2, and topological roles, as well as keystone taxa, were identified based on Zi (within-module connectivity) and Pi (among-module connectivity) values [29]. For PICRUSt functional prediction, the identical set of representative OTU sequences was re-annotated against the Greengenes database (the native compatible database for PICRUSt software, PICRUSt2, v2.4.1) without re-clustering OTUs; this parallel dual-database annotation allowed us to generate functional abundance profiles of microbial communities across different hierarchical levels [30]. Kyoto Encyclopedia of Genes and Genomes (KEGG) Ortholog (KO) functional profiles were generated by comparing Greengenes-based OTU annotations with the KEGG database. The nearest sequenced taxon index (NSTI) was used to evaluate the reliability of the predicted functional profiles.

Redundancy analysis (RDA) was performed to quantify the association between genus-level gut microbial community composition, digestive enzyme activities and fish growth performance indicators. Spearman rank correlation analysis was performed to quantify pairwise associations between dominant gut genera, intestinal morphological parameters, digestive enzyme activities and fish growth indices, visualized as a correlation heatmap.

### 2.3. Data Analysis

All experimental data were subjected to homogeneity of variance testing before statistical analysis. When the variance homogeneity assumptions were satisfied, one-way analysis of variance (ANOVA) was performed using SPSS 19.0 software, followed by Duncan’s multiple range test for multiple comparisons. When the assumption of homogeneity of variance was not met, Dunnett’s T3 test was applied for multiple comparisons. Data are presented as mean ± standard error (SE), and differences were considered statistically significant at *p* < 0.05.

## 3. Results

### 3.1. Growth Performance

After 8 weeks of culture, the growth performance of the eight ornamental fish species is shown in Table 2. The weight gain rate, SGR, and feeding rate of goldfish, golden crucian carp, and crucian carp were significantly higher than those of the other five fish species (*p* < 0.05). However, the FCR of goldfish, golden crucian carp, crucian carp, and sailfin molly was significantly lower than that of the remaining four species (*p* < 0.05). No significant differences were observed in CF and SR among the eight fish species (*p* > 0.05).

### 3.2. Intestinal Digestive Enzyme Activity

The intestinal digestive enzyme activities of the eight fish species are shown in Table 3. Lipase, amylase, and trypsin activities in goldfish, red swordtail, and crucian carp were significantly higher than those observed in the other five fish species (*p* < 0.05).

### 3.3. Histological Structure and Pathological Analysis

#### 3.3.1. Intestine

**Figure 1:**
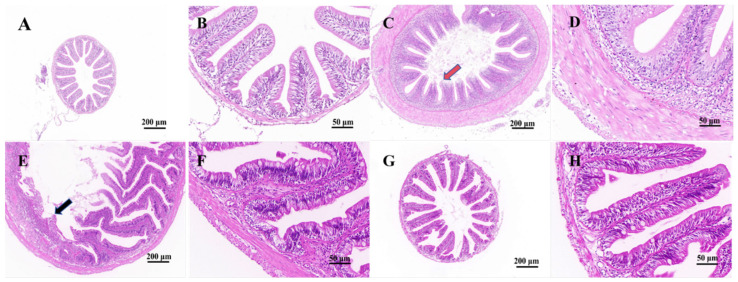

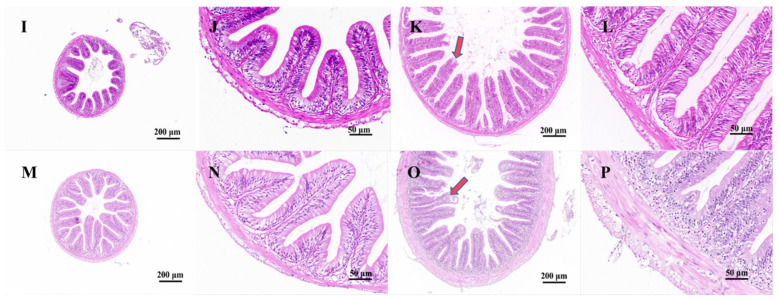
Intestinal histomorphology of eight fish species. Sailfin molly (**A**,**B**), goldfish (**C**,**D**), red swordtail (**E**,**F**), Mickey Mouse platy (**G**,**H**), golden crucian carp (**I**,**J**), platinum mini parrot cichlid (**K**,**L**), sapphire mini parrot cichlid (**M**,**N**), and crucian carp (**O**,**P**). Black arrows indicate intestinal villus atrophy, and red arrows indicate autolysis at the villus tips. Scale bars = 200 μm (**A**,**C**,**E**,**G**,**I**,**K**,**M**,**O**) and 50 μm (**B**,**D**,**F**,**H**,**J**,**L**,**N**,**P**).

Quantitative analysis of intestinal histological sections provided detailed intestinal histomorphological parameters (Table 4; Figure 1). The results showed that goldfish exhibited the greatest muscle layer thickness (117.73 ± 3.26 μm), which was significantly higher than that of the other fish species (*p* < 0.05), followed by crucian carp (59.20 ± 1.65 μm). However, sailfin molly (16.87 ± 2.03 μm) and Mickey Mouse platy (17.30 ± 1.37 μm) possessed the thinnest muscle layers (*p* < 0.05), suggesting a relatively lower degree of intestinal muscular development. Regarding villus height, red swordtail displayed the highest value (553.23 ± 40.51 μm), which was significantly higher than those of the other seven species (*p* < 0.05), followed by crucian carp (321.60 ± 24.52 μm). Golden crucian carp showed the lowest villus height (149.30 ± 10.96 μm) (*p* < 0.05). For villus width, crucian carp (97.93 ± 6.00 μm), platinum mini parrot cichlid (95.60 ± 6.01 μm), and goldfish (97.70 ± 3.98 μm) exhibited significantly better values than the other five fish species (*p* < 0.05), whereas relatively minor variation was observed among the remaining species. Goblet cell density was highest in red swordtail (9449.0 ± 455.1 μm^2^) and Mickey Mouse platy (8558.0 ± 367.5 μm^2^), whereas crucian carp showed the lowest goblet cell density (1718.9 ± 85.0 μm^2^). Analysis of intestinal wall thickness and mucosal layer thickness indicated that red swordtail exhibited the highest intestinal wall thickness (607.55 ± 12.83 μm), followed by crucian carp (395.11 ± 25.19 μm) and goldfish (378.89 ± 25.67 μm). However, golden crucian carp exhibited the thinnest intestinal wall (161.14 ± 21.22 μm) and mucosal layer (154.43 ± 23.12 μm), suggesting a relatively weaker intestinal barrier and a lower nutrient absorption capacity. Red swordtail (594.98 ± 27.67 μm) and crucian carp (389.56 ± 32.56 μm) possessed comparatively thicker mucosal layers.

Histopathological observations further demonstrated marked intestinal villus atrophy and autolysis in red swordtail. Mild autolysis at the villus tips was observed in goldfish, Mickey Mouse platy, platinum mini parrot cichlid, and crucian carp. No obvious pathological changes were detected in the intestinal tissues of sailfin molly and sapphire mini parrot cichlid, indicating relatively strong environmental adaptability and intestinal health status in these species.

#### 3.3.2. Liver

The measurements of hepatocyte perimeter and area in the eight ornamental fish species are presented in Table 5. Among the species examined, Mickey Mouse platy exhibited the smallest hepatocyte perimeter (32.71 ± 3.18 μm), which was significantly lower than that of red swordtail and sapphire mini parrot cichlid (*p* < 0.05). Red swordtail showed the largest hepatocyte perimeter (58.96 ± 5.61 μm), followed by sapphire mini parrot cichlid (53.71 ± 4.89 μm). No significant differences in hepatocyte perimeter were observed among the remaining five fish species (*p* > 0.05). The variation pattern of hepatocyte area was generally consistent with that of hepatocyte perimeter. Mickey Mouse platy exhibited the smallest hepatocyte area (74.78 ± 14.73 μm^2^), whereas red swordtail showed the largest value (244.51 ± 26.92 μm^2^), followed by sapphire mini parrot cichlid (190.55 ± 20.06 μm^2^). No significant differences in hepatocyte area were detected among the remaining five fish species (*p* > 0.05). Hepatocyte perimeter and area displayed clear species-specific characteristics, and the variation trends of these two morphological parameters were highly consistent among the eight ornamental fish species.

**Figure 2:**
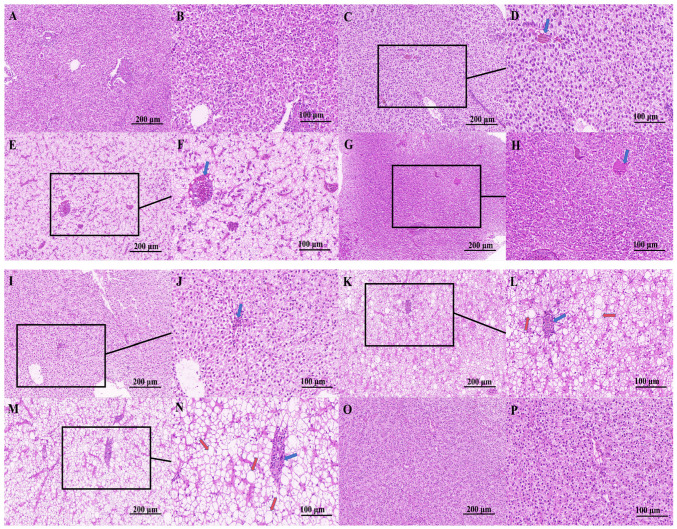
Liver histomorphology of eight fish species. Sailfin molly (**A**,**B**), goldfish (**C**,**D**), red swordtail (**E**,**F**), Mickey Mouse platy (**G**,**H**), golden crucian carp (**I**,**J**), platinum mini parrot cichlid (**K**,**L**), sapphire mini parrot cichlid (**M**,**N**), and crucian carp (**O**,**P**). Blue arrows indicate congestion, and red arrows indicate hepatocyte vacuolization. Scale bars = 200 μm (**A**,**C**,**E**,**G**,**I**,**K**,**M**,**O**) and 100 μm (**B**,**D**,**F**,**H**,**J**,**L**,**N**,**P**).

Additionally, pathological results showed mild hepatocellular congestion in goldfish, red swordtail, Mickey Mouse platy, golden crucian carp, platinum mini parrot cichlid, and sapphire mini parrot cichlid, with extensive vacuolization observed specifically in platinum mini parrot cichlid and sapphire mini parrot cichlid (Figure 2).

### 3.4. Gut Microbiota Analysis

#### 3.4.1. α-Diversity Analysis

**Figure 3:**
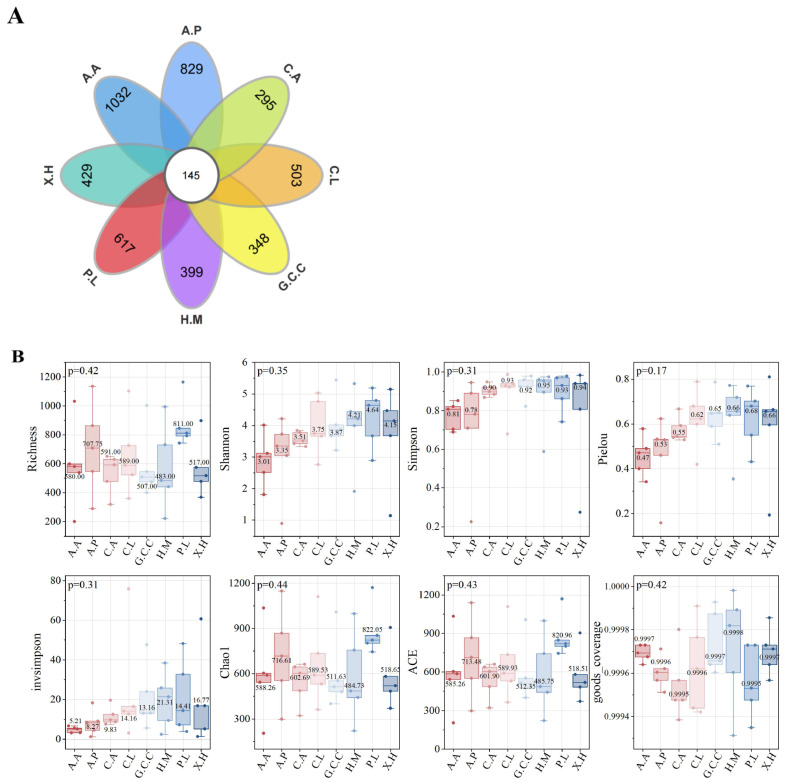
α-Diversity analysis of gut microbiota of eight fish species. (**A**) OTU Venn diagram. (**B**) Diversity indices. P.L., sailfin molly; C.L., goldfish; X.H., red swordtail; H.M., Mickey Mouse platy; G.C.C., golden crucian carp; A.P., platinum mini parrot cichlid; A.A., sapphire mini parrot cichlid; C.A., crucian carp.

The OTU Venn diagram of intestinal microbiota among the eight ornamental fish species is presented in Figure 3A. A total of 145 OTUs were shared among all fish species. Platinum mini parrot cichlid exhibited the highest number of unique OTUs (1032), whereas crucian carp possessed the lowest number of unique OTUs (295).

α-diversity indices were calculated using Mothur software (v.1.48.0, Figure 3B). No significant differences in microbial diversity indices were observed among the eight fish species (*p* > 0.05). However, sailfin molly, red swordtail, and Mickey Mouse platy exhibited relatively higher Shannon indices, indicating comparatively greater microbial diversity. Furthermore, sailfin molly and platinum mini parrot cichlid showed relatively higher Chao1 and ACE indices than the other six fish species, suggesting increased microbial richness.

#### 3.4.2. β-Diversity Analysis

**Figure 4:**
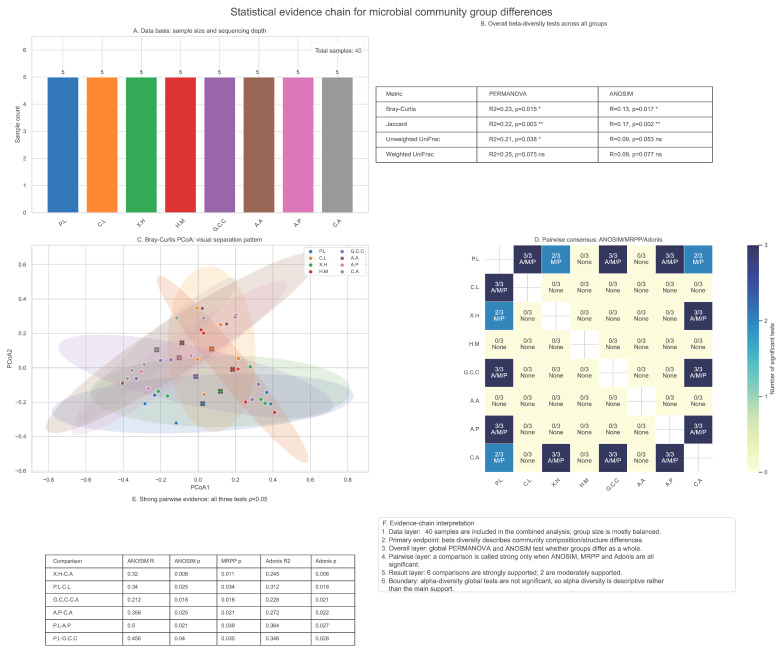

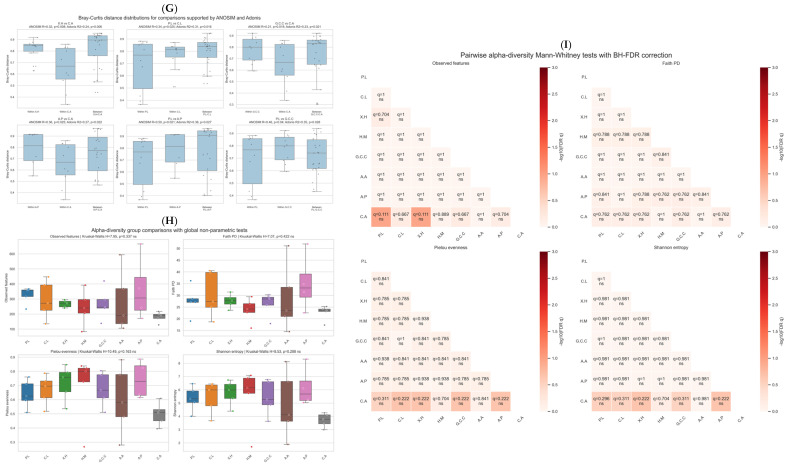
Statistical evidence chain for microbial community group differences. P.L., sailfin molly; C.L., goldfish; X.H., red swordtail; H.M., Mickey Mouse platy; G.C.C., golden crucian carp; A.P., platinum mini parrot cichlid; A.A., sapphire mini parrot cichlid; C.A., crucian carp. * significant difference (0.01 < *p* < 0.05), ** highly significant difference (*p* < 0.01), ns not significant (*p* ≥ 0.05).

Statistical evidence chain for microbial community group differences (Figure 4). Based on 16S rDNA sequencing results, this study first conducted beta diversity analysis of the microbial community structure across 40 samples, and employed PERMANOVA, ANOSIM, and MRPP to perform permutation tests for inter-group differences. Overall test results showed that differences in community structure among groups were statistically supported under both Bray–Curtis and Jaccard distance metrics. Further pairwise comparisons revealed that contrasts such as P.L-C.L, P.L-G.C.C, P.L-A.P, X.H-C.A, G.C.C-C.A, and A.P-C.A achieved *p* < 0.05 in all three tests—ANOSIM, MRPP, and Adonis—indicating robust statistical support for microbial community differences between these groups. Global tests of alpha diversity did not reach significant levels; therefore, their results were primarily used to describe trends in diversity among groups, while the core evidence for community structure differences came from the beta diversity distance matrix and consistent findings across multiple tests.

#### 3.4.3. Microbial Composition Analysis

**Figure 5:**
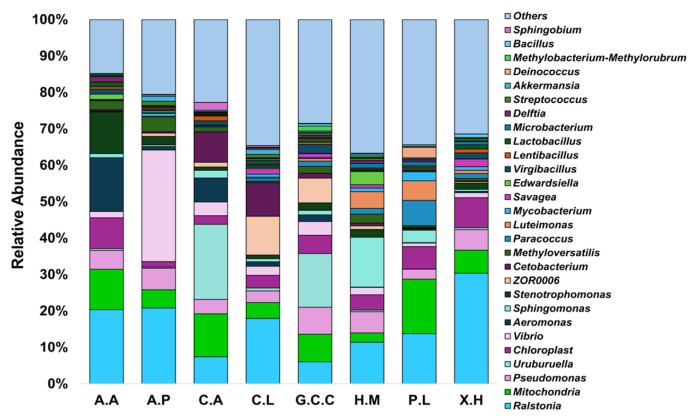
Composition of gut microbiota at the genus level. P.L., sailfin molly; C.L., goldfish; X.H., red swordtail; H.M., Mickey Mouse platy; G.C.C., golden crucian carp; A.P., platinum mini parrot cichlid; A.A., sapphire mini parrot cichlid; C.A., crucian carp.

Representative OTU sequences were taxonomically classified using the RDP classifier Bayesian algorithm. The relative abundance distribution of intestinal microbial communities at the genus level is shown in Figure 5. *Ralstonia* and *Pseudomonas* within Proteobacteria were identified as common dominant genera among the eight ornamental fish species. Furthermore, *Aeromonas* was the dominant genus in sapphire mini parrot cichlid and crucian carp, whereas *Vibrio* was dominant in platinum mini parrot cichlid. *Uruburuella* showed relatively high abundance in crucian carp, golden crucian carp, and Mickey Mouse platy, while *Edwardsiella* was enriched in Mickey Mouse platy. *Cetobacterium* was identified as the dominant genus in crucian carp and goldfish, whereas *Paracoccus* was dominant in sailfin molly.

#### 3.4.4. Differential Abundance Species Analysis

**Figure 6:**
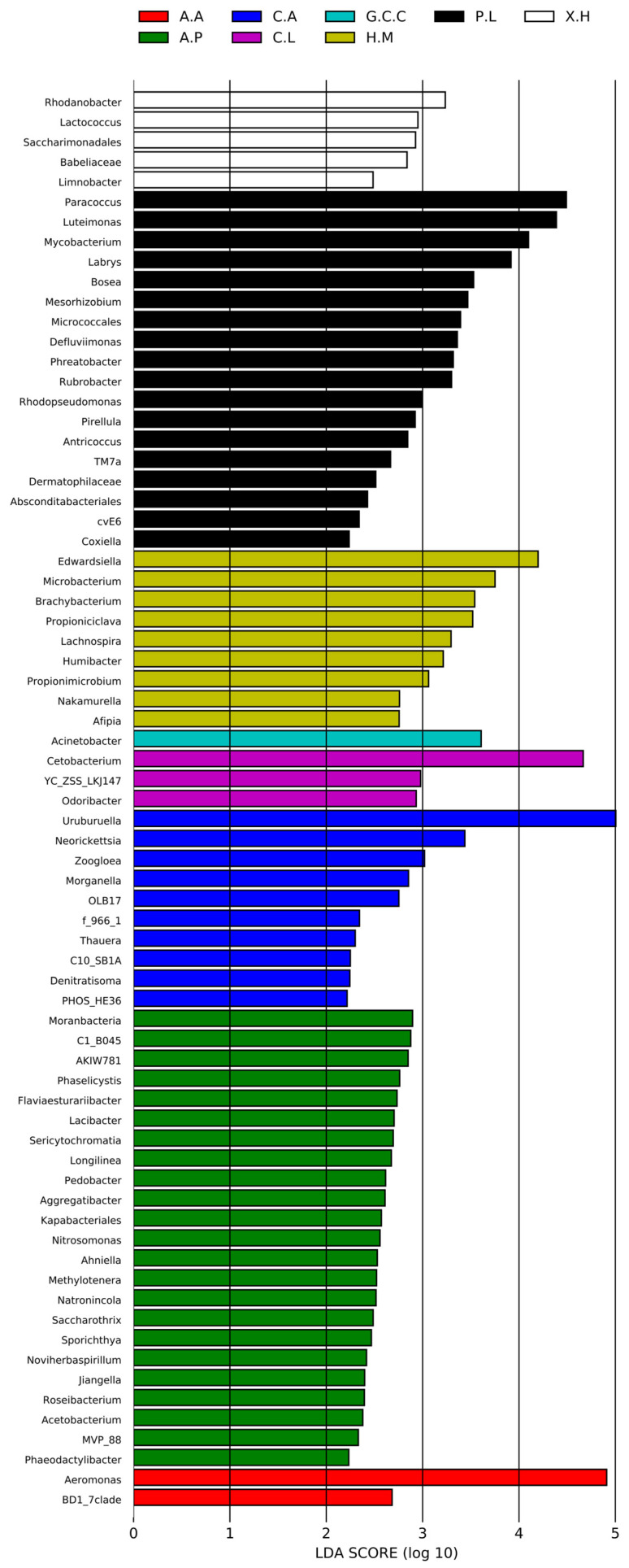
Differential species analysis of gut microbiota. P.L., sailfin molly; C.L., goldfish; X.H., red swordtail; H.M., Mickey Mouse platy; G.C.C., golden crucian carp; A.P., platinum mini parrot cichlid; A.A., sapphire mini parrot cichlid; C.A., crucian carp.

Differential microbial taxa among the eight fish species were identified by LEfSe analysis using the Kruskal–Wallis H test, followed by linear discriminant analysis (LDA) to estimate effect size (Figure 6). The contribution of significantly different taxa to microbial community variation was then visualized based on LDA scores (LDA > 2). The results demonstrated that the relative abundances of *Rhodanobacter* and *Lactococcus* were significantly enriched in red swordtail, whereas *Paracoccus* and *Luteimonas* were significantly enriched in sailfin molly. *Cetobacterium* abundance was significantly increased in goldfish. The relative abundances of potentially pathogenic genera, including *Edwardsiella, Aeromonas*, and *Acinetobacter*, were significantly elevated in Mickey Mouse platy, sapphire mini parrot cichlid, and golden crucian carp, respectively.

#### 3.4.5. Phylogenetic Analysis

**Figure 7:**
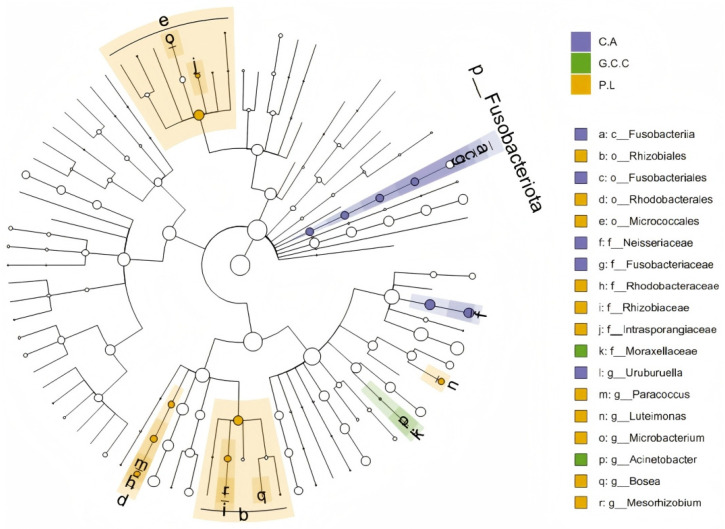
Community phylogenetic distribution of gut microbiota. C.A., crucian carp; G.C.C., golden crucian carp; P.L., sailfin molly.

Based on the Bray–Curtis distance matrix, a phylogenetic tree of intestinal microbial communities from the eight ornamental fish species was constructed using the UPGMA (Figure 7). The results demonstrated a close phylogenetic relationship between the gut microbial communities of sailfin molly and Mickey Mouse platy, suggesting that these two species may share similar community composition or exhibit host-associated clustering patterns. However, the gut microbial communities of crucian carp and golden crucian carp showed relatively greater phylogenetic distance, indicating significant divergence in microbial community structure during long-term adaptation to distinct ecological niches and habitats. The phylogenetic analysis also revealed a relatively close relationship between the microbial communities of sailfin molly and crucian carp, whereas considerable differences were observed between the microbial communities of red swordtail and Mickey Mouse platy.

**Figure 8:**
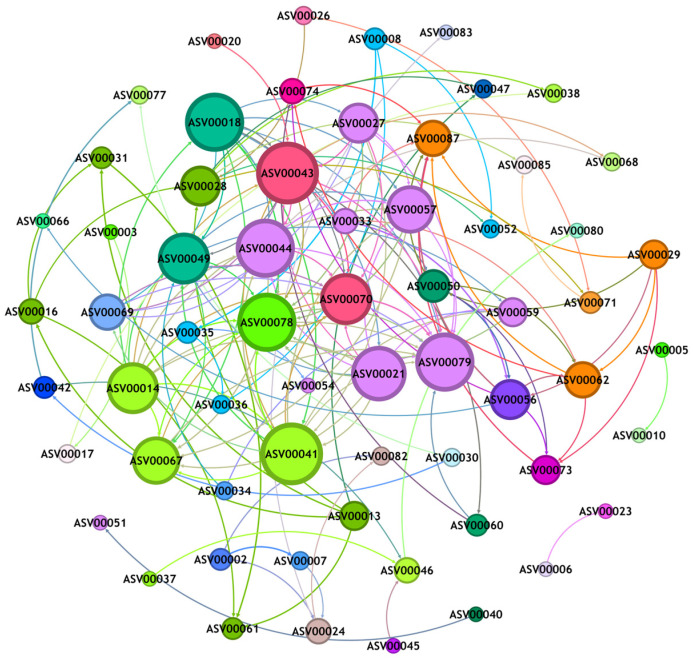
Co-occurrence network of intestinal microbiota in eight ornamental fish species.

The co-occurrence network analysis of gut microbiota at the ASV level is presented in Figure 8. The network revealed marked functional differentiation among ornamental fish species in key metabolic pathways, including carbohydrate metabolism, lipid metabolism, and amino acid metabolism. Several ASVs, including ASV00044, ASV00041, ASV00043, and ASV00078, exhibited relatively large node sizes, indicating high abundance and central importance within the intestinal microbial communities of the eight fish species. These ASVs likely represent core symbiotic microorganisms that participate in the regulation of host intestinal health and growth. Among these core ASVs, the connections between ASV00044 and ASV00078, as well as between ASV00044 and ASV00041, displayed relatively greater edge thickness, suggesting strong co-occurrence relationships and possible synergistic ecological functions under similar environmental conditions. Furthermore, ASV00027, ASV00070, and ASV00095 also showed strong co-occurrence patterns, suggesting potential similarities in ecological function or coordinated responses to environmental factors within the gut microecosystem. The microbial co-occurrence network could be divided into several distinct clusters. A green cluster located in the lower-left region of the network was centered on ASV00044, ASV00041, and ASV00036. A blue cluster in the upper-right region was mainly composed of ASV00008 and ASV00010, whereas the purple and pink clusters in the central region were dominated by ASV00027 and ASV00070, respectively. ASVs within the same cluster exhibited relatively high similarity in metabolic pathways, ecological functions, or environmental responses, reflecting ecological niche differentiation among different host species and microbial groups.

#### 3.4.6. Functional Prediction Analysis

**Figure 9:**
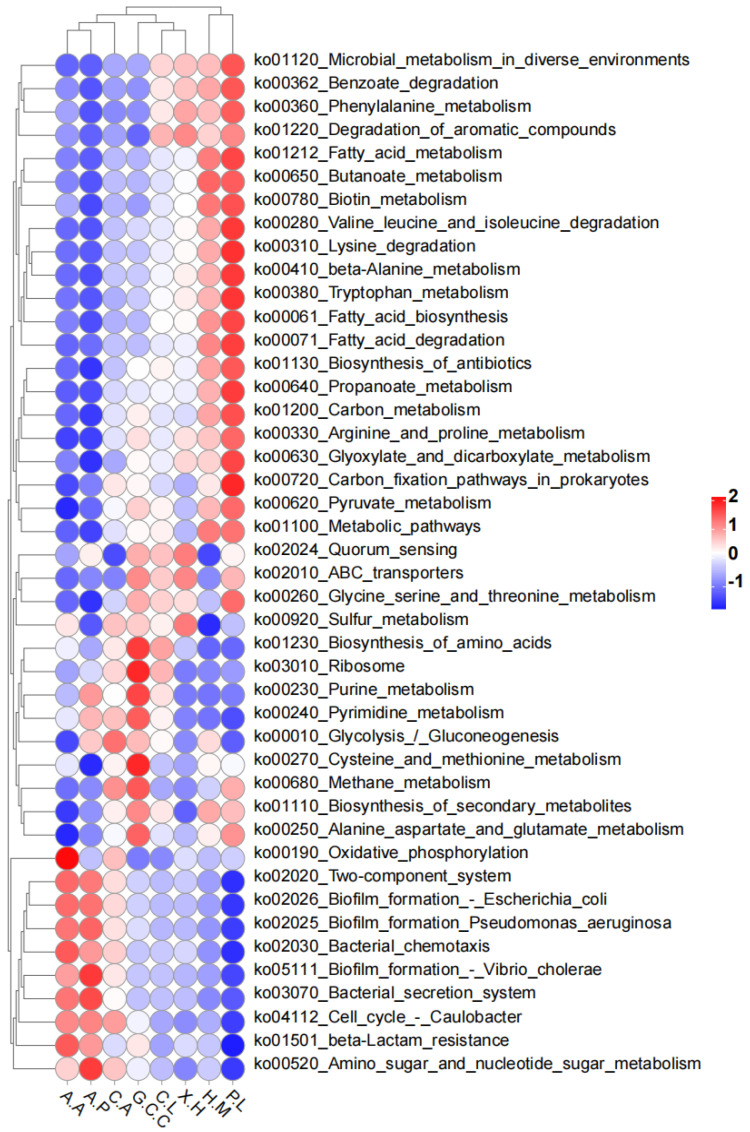
Functional prediction analysis of gut microbial communities. P.L., sailfin molly; C.L., goldfish; X.H., red swordtail; H.M., Mickey Mouse platy; G.C.C., golden crucian carp; A.P., platinum mini parrot cichlid; A.A., sapphire mini parrot cichlid; C.A., crucian carp.

Functional prediction of intestinal microbial communities from eight ornamental fish species was performed using PICRUSt, and differences in metabolic functions were analyzed using KEGG pathways (Figure 9). The mean weighted NSTI value of all samples was confirmed to be 0.38. Although this low NSTI value reflects that most microbial taxa share close phylogenetic relatedness with sequenced reference genomes, a small subset of microbes still exhibit relatively large phylogenetic distances from available reference sequences. In addition, the taxonomic annotation and functional prediction of this study relied on dual databases (SILVA and Greengenes) with corresponding database conversion procedures. For the above reasons, all KEGG functional predictions derived from PICRUSt should be interpreted cautiously. The PICRUSt outputs are only exploratory functional inferences and cannot be equated with actual metagenomic measurement data.

The PICRUSt outputs indicated potential functional divergence among the gut microbiota of different ornamental fish species, particularly in pathways associated with metabolism, signal transduction, and cellular processes. These predicted functional profiles appeared to correlate with host physiological status and growth performance. The sailfin molly and Mickey Mouse platy displayed the most prominent enrichment of metabolic pathways, including amino acid metabolism modules such as phenylalanine metabolism (ko00360), tryptophan metabolism (ko00380), and arginine and proline metabolism (ko00330), alongside fatty acid metabolism (ko01212), fatty acid biosynthesis (ko00061), biotin metabolism (ko00780), and pyruvate metabolism (ko00620). These predictions may reflect potential advantages in lipid metabolic regulation, immune-associated metabolism, detoxification, and cell proliferation for the two species.

Crucian carp and golden crucian carp showed elevated predicted activity in ribosome metabolism (ko03010), amino acid biosynthesis (ko01230), methane metabolism (ko00680), glycolysis/gluconeogenesis (ko00010), and pyrimidine metabolism (ko00240). Such functional patterns may support enhanced nutrient digestion, energy acquisition, glucose and osmotic homeostasis, and cell proliferation, which align with our histomorphological observations of well-developed intestinal villi, thick mucosal layers, and robust digestive and absorptive capacity in crucian carp. Similarly, golden crucian carp was predicted to be enriched in purine metabolism (ko00230), cysteine and methionine metabolism (ko00270), alanine, aspartate, and glutamate metabolism (ko00250), and ATP-binding cassette (ABC) transporter pathways (ko02010).

Goldfish exhibited relatively high predicted enrichment in quorum sensing (ko02024), ABC transporters, ribosome metabolism, and amino acid biosynthesis, which may imply strengthened microbial intercellular communication and transmembrane transport within its intestinal community. Platinum mini parrot cichlid and sapphire mini parrot cichlid were mainly characterized by predicted enrichment of cellular process pathways, including the cell cycle (ko04112) and bacterial secretion system (ko03070), as well as pathogenic biofilm formation pathways such as Escherichia coli biofilm formation (ko02026), Vibrio biofilm formation (ko02025), and Pseudomonas aeruginosa biofilm formation (ko05111). Enrichment of amino sugar and nucleotide sugar metabolism (ko00520) was also detected, which may suggest active microbial cellular metabolism and immune-related responses. In addition, the sapphire mini parrot cichlid displayed relatively high predicted activity in oxidative phosphorylation (ko00190).

The red swordtail had elevated predicted functional activity in quorum sensing, phenylalanine metabolism, ABC transporters, and sulfur metabolism (ko00920). These predicted profiles hint that the intestinal microbiota of red swordtails may modulate virulence-related factors, immune processes, and host nutrient absorption and metabolism, consistent with our morphological observation of a robust mucus-based intestinal protective barrier in this species.

#### 3.4.7. Associations of Gut Microbiota with Digestive Enzymes and Growth Traits

**Figure 10:**
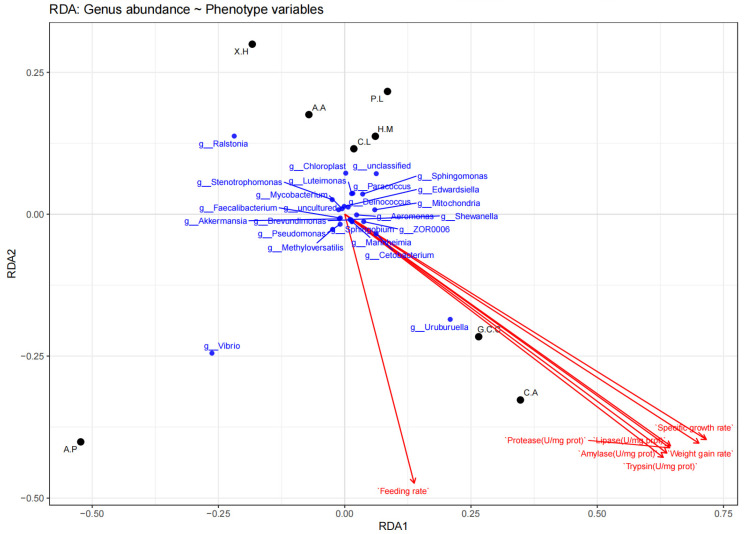
Correlation between intestinal microbiota, digestive enzyme activities and growth performance. Black dots = fish samples, blue text = bacterial genera, red arrows = growth and digestive enzyme phenotypic indicators. The length and included angle of red arrows respectively represent correlation magnitude and correlation direction between variables.

Correlation between intestinal microbiota, digestive enzyme activities and growth performance was presented in Figure 10. The arrows for protease, lipase, amylase, trypsin, weight gain rate, and specific growth rate are almost perfectly aligned, indicating a strong consistency in the trends of these growth and digestive phenotypes. The feeding rate arrow points in the exact opposite direction, showing a negative correlation with digestive enzyme activities. Right-side genera (*Sphingomonas*, *Edwardsiella*, *Shewanella*, *Uruburuella*) are significantly positively correlated with high digestive enzyme activity and superior growth performance. Left-side genera (*Vibrio*, *Ralstonia*, *Akkermansia*, *Stenotrophomonas*) are positively associated with feeding rate but negatively correlated with digestive enzyme activities. Samples from G.C and C.A cluster in the quadrant of high digestive enzyme activity. A.P samples fall into an area enriched with *Vibrio*, characterized by high feeding rates but poor growth; X.H, A.A, P.L, H.M, and C.L are distributed centrally.

**Figure 11:**
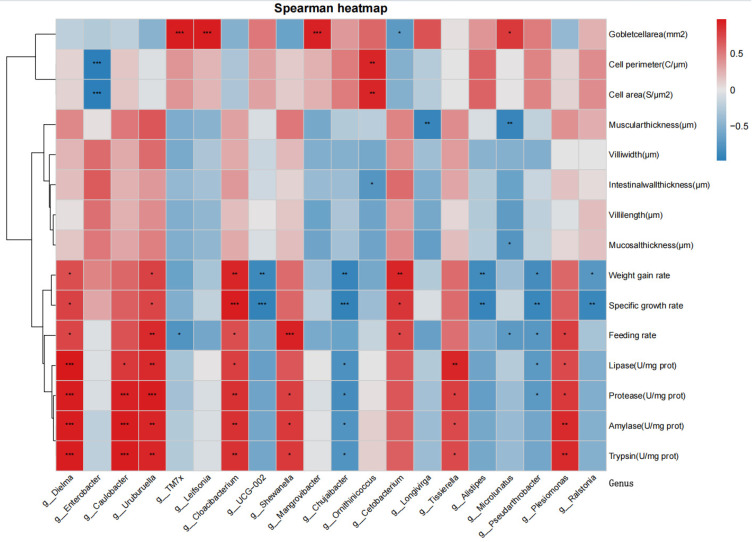
Spearman correlation between key intestinal taxa, intestinal morphology, digestive enzyme activity and growth performance. * *p* < 0.05 (significant), ** *p* < 0.01 (highly significant), *** *p* < 0.001 (extremely highly significant).

Spearman correlation between key intestinal taxa, intestinal morphology, digestive enzyme activity and growth performance was presented in Figure 11. The higher the abundance of bacteria such as *Dielma*, *Enterobacter*, *Caulobacter*, *Uruburuella*, *Leifsonia*, and *Cloacibacterium*, the more significantly the digestive enzyme activity, weight gain, and specific growth rate increase simultaneously. The higher the abundance of bacteria such as *UCG-002*, *Mangrovibacter*, *Chujaibacter*, *Alistipes, Microlunatus*, *Pseudarthrobacter*, and *Ralstonia*, the more significantly the digestive enzymes and growth rate decrease. The feed rate is negatively correlated with most growth-promoting bacteria and positively correlated with inhibitory bacteria, indicating that eating a lot does not necessarily mean growing fast. The bacterial community mediates the differences in digestive and absorption efficiency.

The size of goblet cells, the size of epithelial cells (area/perimeter), and Leifsonia, Cetobacterium, and Tissierella are significantly positively correlated. The thickness of the muscular layer, the size of villi, the thickness of the intestinal wall mucosa, and Alistipes, Microlunatus are negatively correlated. Among them, Uruburuella has a strong positive correlation with all digestive enzymes and growth indicators, and a negative correlation with all adverse intestinal morphology indicators. Ralstonia has a significant negative correlation with all digestive enzymes and growth rate, Shewanella has a positive correlation with the feeding rate, and a weak negative correlation with growth indicators.

## 4. Discussion

### 4.1. Intestinal Tissue Morphology and Fish Health

The integrity of intestinal structure not only directly or indirectly affects the physiological functions of fish but also plays a key role in maintaining fish health. As the primary site of nutrient digestion and absorption, intestinal villus height and width determine the available absorptive surface area and, thus, affect nutrient uptake efficiency [31,32]. In this study, red swordtail and crucian carp exhibited higher villus height and width than the other six ornamental fish species. These structural characteristics provide a favorable morphological basis for efficient nutrient absorption and are generally consistent with the relatively high intestinal digestive enzyme activities observed in these species. However, despite well-developed villi, red swordtail exhibited severe intestinal pathology, likely reducing nutrient utilization efficiency and ultimately leading to poor growth performance. In addition to genetic and developmental determinants, external factors such as dietary composition and aquaculture environment can alter intestinal villus morphology by regulating the proliferation and differentiation of intestinal epithelial cells, affecting nutrient absorption efficiency [33]. A thicker intestinal muscular layer generally provides stronger peristaltic capacity, allowing feed to be transported, digested, and absorbed more efficiently within a relatively limited intestinal length [32]. Goldfish and crucian carp possessed relatively thicker intestinal muscular layers, indicating stronger intestinal peristalsis. This characteristic may be associated with the absence of a true stomach and the more herbivorous feeding characteristics of these species. Previous studies have also demonstrated that a well-developed intestinal structure facilitates the colonization of beneficial microorganisms and promotes intestinal development and digestive function [34]. These results suggest that under the culture conditions used in this study, dietary supplementation with probiotics may further improve intestinal health and digestive capacity in ornamental fish.

Goblet cells secrete mucus that protects the intestinal epithelium against mechanical damage and pathogenic microorganism invasion. The relatively larger goblet cell areas observed in red swordtail, sailfin molly, and Mickey Mouse platy suggest stronger mucus secretion capacity and enhanced intestinal mucosal defense [35]. Intestinal wall thickness reflects intestinal structural robustness and the ability to adapt to environmental fluctuations. Red swordtail and crucian carp exhibited relatively thicker intestinal walls, indicating stronger intestinal structural integrity and potentially higher tolerance to environmental stress [36]. Previous studies on the intestinal microbial diversity of common carp (*Cyprinus carpio*) have demonstrated that intestinal structural integrity and function are closely associated with fish health and the stability of the microbial community [37,38]. Similarly, studies investigating intestinal microbiota during the early developmental stages of large yellow croaker (*Larimichthys crocea*) reported that intestinal microbial composition and function are closely related to intestinal morphology and that healthy intestinal structures contribute to the maintenance of stable microbial communities and improved host health [39].

Intestinal structural integrity represents a fundamental basis for maintaining normal physiological function, health status, and adaptability to aquaculture environments. Significant species-specific differences were observed among ornamental fish species in intestinal villus height, muscular layer thickness, goblet cell area, and intestinal wall thickness. These morphological indicators are closely associated with nutrient digestion and absorption, intestinal motility, mucosal protection, and resistance to environmental stress. Fish intestinal health is jointly regulated by genetic background, dietary composition, and aquaculture environmental conditions. At the same time, intestinal morphology and microbial communities exhibit a close bidirectional relationship. A healthy intestinal structure provides specific ecological niches that support the colonization of adapted microbial taxa, promoting host–microbe co-development. In turn, a stable intestinal microbial community helps maintain intestinal integrity and physiological function. Therefore, optimization of feed formulations, including supplementation with probiotics or plant-derived additives, together with improvements in aquaculture environmental management, may enhance intestinal morphology and microbial diversity, improving fish health, growth performance, and aquaculture productivity.

### 4.2. Liver Tissue Morphology and Fish Health

Histological analysis of liver tissues demonstrated significant interspecific differences in hepatocyte morphology, including cell perimeter and cell area, indicating substantial variation in liver structural characteristics and physiological adaptability among ornamental fish species. Red swordtail and sapphire mini parrot cichlid exhibited relatively larger hepatocyte perimeter and area compared with the other fish species. Larger hepatocyte size is generally associated with increased metabolic activity and may support more efficient nutrient absorption, transformation, and storage [40]. Mickey Mouse platy exhibited significantly smaller hepatocyte perimeter and area than the other seven species, suggesting relatively limited hepatic metabolic capacity, which may contribute to reduced physiological performance and overall health status.

Histopathological observations further revealed significant differences in liver health among the eight ornamental fish species under identical aquaculture conditions. Except for sailfin molly and crucian carp, the remaining six fish species exhibited mild hepatic congestion and tissue injury, indicating that these species experienced relatively higher physiological stress under the same culture environment. Such pathological changes may be associated with local circulatory disturbances caused by excessive hepatic detoxification burden [41]. In the platinum mini parrot cichlid and sapphire mini parrot cichlid, large numbers of lipid droplet vacuoles were observed within hepatocytes. Previous studies have indicated that lipid vacuolation may result from high-lipid or high-carbohydrate diets, deficiencies in nutrients associated with lipid metabolism, or environmental stress and toxin exposure [42]. Under the experimental conditions of this study, vacuole formation may have been associated with abnormal lipid accumulation or insufficient availability of key nutrients involved in lipid metabolism. These pathological alterations may impair normal hepatic physiological function and potentially increase susceptibility to liver dysfunction. The liver tissues of sailfin molly and crucian carp showed no obvious lesions, and their hepatocyte perimeters and areas remained within a relatively moderate range. This favorable histological condition suggests that these species were able to maintain relatively stable liver physiological function under the same aquaculture conditions, indicating comparatively strong environmental adaptability.

The eight ornamental fish species exhibited clear interspecific differences in hepatocyte morphology and pathological characteristics. Hepatocyte perimeter and area may directly reflect the basal metabolic potential of the liver. Species with relatively larger hepatocyte morphological parameters generally showed stronger hepatic metabolic activity, although this was accompanied by mild congestion and lipid accumulation. Species with smaller hepatocyte parameters exhibited relatively weaker metabolic potential and more pronounced hepatic pathological changes, indicating reduced adaptability to environmental conditions. Fish species with moderate hepatocyte morphological characteristics exhibited comparatively normal liver physiology and stronger environmental adaptability. Therefore, hepatocyte morphological parameters, including perimeter and area, may serve as useful histological indicators for evaluating liver health and environmental adaptability in ornamental fish. These indicators may also provide a theoretical basis for selecting stress-resistant ornamental fish varieties, optimizing feed formulations, and improving aquaculture management strategies.

### 4.3. Gut Microbiota and Fish Health

The 16S rRNA sequencing analysis demonstrated that although no significant differences were detected in α-diversity among the eight ornamental fish species, the Shannon index, Chao1 index, and ACE index of the sailfin molly exhibited an increasing trend, indicating relatively higher microbial richness, diversity, and community evenness. Previous studies have shown that highly diverse intestinal microbial communities facilitate the proliferation of beneficial microorganisms and short-chain fatty acid (SCFA)-producing bacteria, improving nutrient absorption, immune function, and disease resistance in the host [43]. In this study, the relative abundance of *Paracoccus* was significantly enriched in the intestine of the sailfin molly. *Paracoccus* has been reported to synthesize docosahexaenoic acid (DHA) and eicosapentaenoic acid (EPA), while also contributing to microbial community stability, antioxidant capacity, and maintenance of intestinal barrier integrity [44,45]. These characteristics may partially explain the relatively favorable feed conversion ratio and liver histological status observed in the sailfin molly. Additionally, the capacity for DHA and EPA synthesis may contribute to enhanced vitality and improved body coloration in ornamental fish [46,47]. Furthermore, the relative abundances of *Lactococcus* and *Cetobacterium* were significantly increased in the red swordtail and goldfish, respectively. *Lactococcus* can reduce intestinal pH, inhibit the proliferation of harmful microorganisms, and promote carbohydrate metabolism and intestinal immune function. *Cetobacterium* is capable of producing acetate, regulating amino acid metabolism and glucose homeostasis, and strengthening intestinal barrier and immune functions [48,49].

The exploratory KEGG functional profiles inferred via PICRUSt provided auxiliary supporting evidence for the above observations, with predicted enrichment of phenylalanine metabolism, glycine, serine and threonine metabolism, and ABC transporter pathways detected in these fish species. Notably, the reliability of such predicted functional profiles is constrained by incomplete coverage of sequenced reference genomes and the dual-taxonomy annotation pipeline combining SILVA and Greengenes databases; accordingly, all functional interpretations herein are treated as preliminary exploratory inferences.

In terms of microbial community differentiation, the robust statistical basis confirming intergroup structural divergence relies on consistent outputs from Beta diversity matrices and multiple complementary permutation tests (PERMANOVA, ANOSIM, MRPP and Adonis), as detailed in Section 3. Both global and pairwise comparison results collectively verified stable significant community dissimilarities across six group pairs, whereas non-significant global Alpha diversity indices and LEfSe biomarkers only serve as secondary descriptive evidence rather than decisive statistical proof for grouping differences. This tiered statistical framework strengthens the credibility of our community structure findings, while the PICRUSt functional predictions remain tentative supplementary reference due to inherent analytical limitations.

Potentially pathogenic genera, including *Edwardsiella, Aeromonas*, and *Acinetobacter*, were significantly enriched in the intestines of Mickey Mouse platy, sapphire mini parrot cichlid, and golden crucian carp, respectively. *Edwardsiella* is recognized as one of the major pathogenic bacteria causing septicemia and enteritis in fish by disrupting intestinal barrier integrity. *Aeromonas* can induce intestinal microbial dysbiosis and produce virulence-associated toxins, including hemolysins and enterotoxins, which damage intestinal mucosal tissues. Although *Acinetobacter* is generally considered less virulent than *Edwardsiella* and *Aeromonas*, it may still induce disease under certain environmental or physiological conditions [50,51]. RDA and Spearman correlation analyses jointly illustrated the regulatory cascade linking intestinal microbiota, gut morphology, digestive function and growth traits across the eight fish species. In this study, interspecific differences in gut microbiota remodeled intestinal morphological structure and modulated digestive enzyme output, ultimately driving distinct growth efficiency among tested fish species. Functional prediction analysis suggested that *Edwardsiella* enrichment in Mickey Mouse platy was associated with increased arginine and tryptophan metabolism, which may activate target of rapamycin (TOR)-related immune responses and improve host immune defense and disease resistance [52]. In sapphire mini parrot cichlid enriched with *Aeromonas*, pathways associated with pathogenic biofilm formation, amino sugar and nucleotide sugar metabolism, and oxidative phosphorylation were significantly upregulated. These results suggest that a significant proportion of fish energy may be redirected toward immune defense under persistent pathogenic pressure, potentially contributing to reduced growth performance despite relatively high feed intake. Increased oxidative phosphorylation activity may also promote ROS production, which could contribute to hepatocyte vacuolization observed in sapphire mini parrot cichlid [53]. Golden crucian carp enriched with *Acinetobacter* exhibited relatively high functional activity in ribosome metabolism, glycolysis/gluconeogenesis, purine metabolism, and amino acid biosynthesis pathways. From PICRUSt predictive results, the altered metabolic pathways may represent putative trends of amplified energy provision and upregulated biosynthesis of immune factors. Further empirical verification is required to confirm whether these pathway shifts are able to facilitate host anti-pathogen defense performance [54].

It should be noted that despite unified formulated feed and body-weight-proportional feeding rates of 3–5% for all eight fish species, extreme gaps in initial body weight and species-specific trophic habits caused inconsistent nutritional adaptation to the same diet. This acts as a critical confounding factor alongside inherent phylogenetic and physiological disparities across taxa. For this reason, all interspecies comparisons are interpreted conservatively; phenotypic variations in intestinal structure, digestive enzymes, liver histology and gut microbiota cannot be ascribed only to intrinsic biological traits due to divergent feed adaptability. Follow-up controlled trials with standardized initial body size and species-matched feed formulas are required to disentangle these complex interacting variables and verify interspecific biological mechanisms more precisely.

In summary, under identical aquaculture conditions, the intestinal microbial communities of different ornamental fish species exhibited strong species-specific characteristics. These differences are likely associated with host physiological requirements and environmental adaptation strategies. Core intestinal microbial genera displayed divergent distribution patterns across fish species, and are predicted to undertake species-specific nutritional and metabolic functional roles. Meanwhile, the gut microbiota is presumed to modulate host immune responses and sustain intestinal homeostasis via diverse metabolic and immune-associated pathways; definitive functional verification will be implemented in follow-up experiments to confirm these regulatory mechanisms.

## 5. Conclusions

This study systematically analyzed the composition and predicted functional profiles of the intestinal microbiota across eight ornamental fish species. The results suggest that even under identical rearing conditions, different fish species assemble divergent gut microbial communities that are plausibly linked to growth performance and intestinal health status. Molly presented relatively high gut microbial alpha diversity and richness, which may facilitate the proliferation of the potential probiotic bacterium *Paracoccus*. Furthermore, PICRUSt prediction revealed enriched metabolic pathways (amino acid, fatty acid, vitamin metabolism and antibiotic biosynthesis) in molly, implying putative robust lipid metabolism and immune potential. Goldfish and crucian carp possessed intact intestinal morphological structures and efficient digestive phenotypes, accompanied by elevated predicted carbohydrate and pyrimidine metabolism, which could hypothetically assist stress adaptation. Golden crucian carp showed inferior intestinal tissue morphology yet sustained normal growth, possibly via upregulated predicted amino acid biosynthesis, purine and pyrimidine metabolic pathways. Platinum and sapphire mini parrot cichlids tended to display signs of hepatic stress with cellular vacuolization. The sapphire mini parrot cichlid may potentially counteract opportunistic pathogens (*Aeromonas*, *Escherichia coli*, *Vibrio*, *Pseudomonas*) by boosting energy generation through activated oxidative phosphorylation, as predicted. Swordtail exhibited favourable intestinal structural defense and hepatic metabolic traits, while Mickey Mouse platy might mitigate *Edwardsiella* infection risk through modulated arginine/tryptophan metabolism, stimulated TOR signaling and enhanced predicted antibiotic biosynthesis. All above outcomes are observational sequencing-based deductions without direct functional validation; these exploratory findings offer preliminary reference for optimizing rearing strategies, health maintenance and culture efficiency in ornamental fish farming, and targeted functional trials will be conducted in future work to verify these inferred mechanisms.

## Figures and Tables

**Table 1:** Comparison of eight ornamental fish families, genera, and species.

Fish	Families	Genera	Species	IBL (cm)	IBW (g)
Sailfin molly	Poeciliidae	*Poecilia*	*Poecilia latipinna*	5.50 ± 0.50	2.98 ± 0.02
Goldfish	Cyprinidae	*Carassius*	*Carassius auratus*	11.50 ± 0.50	27.27 ± 0.23
Red swordtail	Poeciliidae	*Xiphophorus*	*Xiphophorus hellerii*	8.00 ± 0.20	9.23 ± 0.17
Mickey Mouse platy	Poeciliidae	*Xiphophorus*	*Xiphophorus hellerii × Xiphophorus maculatus*	3.78 ± 0.50	0.93 ± 0.12
Golden crucian carp	Cyprinidae	*Carassius*	*Carassius auratus* red var. ♂ × *Cyprinus carpio* L. mirror ♀	12.33 ± 0.50	33.56 ± 0.28
Platinum mini parrot cichlid	Cichlidae	Taxonomic species unconfirmed	Taxonomic species unconfirmed	7.70 ± 0.50	7.36 ± 0.13
Sapphire mini parrot cichlid	Cichlidae	Taxonomic species unconfirmed	Taxonomic species unconfirmed	7.45 ± 0.50	8.23 ± 0.22
Crucian carp	Cyprinidae	*Carassius*	*Carassius carassius*	11.90 ± 0.50	30.30 ± 0.22

IBL, initial body length; IBW, initial body weight. Platinum mini parrot cichlid and sapphire mini parrot cichlid are artificially hybrid ornamental strains belonging to the family Cichlidae. No systematic taxonomic study has confirmed their valid genus and species assignments. The common name “parrot cichlid” is only a commercial aquarium term and cannot be treated as a formal taxonomic species name.

**Table 2:** Growth performance indicators of different ornamental fish species.

Items	ICF (g/cm^3^)	WGR (%)	FCF (g/cm^3^)	SGR (%/d)	FR (%)	FCR	SR (%)
Sailfin molly	1.80 ± 0.08	70.93 ± 2.13 ^c^	1.77 ± 0.06	0.95 ± 0.01 ^c^	1.85 ± 0.05 ^d^	1.98 ± 0.01 ^b^	100 ± 0.00
Goldfish	1.80 ± 0.07	100.13 ± 0.33 ^a^	1.84 ± 0.06	1.23 ± 0.03 ^a^	2.33 ± 0.01 ^a^	1.96 ± 0.01 ^b^	100 ± 0.00
Red swordtail	1.80 ± 0.09	68.95 ± 0.20 ^c^	1.78 ± 0.01	0.94 ± 0.00 ^c^	2.20 ± 0.10 ^b^	2.40 ± 0.01 ^a^	100 ± 0.00
Mickey Mouse platy	1.80 ± 0.10	77.90 ± 3.93 ^b^	1.72 ± 0.06	1.02 ± 0.07 ^b^	2.06 ± 0.08 ^c^	2.07 ± 0.09 ^b^	100 ± 0.00
Golden crucian carp	1.80 ± 0.08	100 ± 0.00 ^a^	1.86 ± 0.04	1.24 ± 0.00 ^a^	2.33 ± 0.00 ^a^	1.96 ± 0.01 ^b^	100 ± 0.00
Platinum mini parrot cichlid	1.80 ± 0.08	70.61 ± 0.72 ^c^	1.79 ± 0.06	0.93 ± 0.02 ^c^	2.20 ± 0.02 ^b^	2.41 ± 0.07 ^a^	100 ± 0.00
Sapphire mini parrot cichlid	1.80 ± 0.09	68.94 ± 2.14 ^c^	1.79 ± 0.06	0.95 ± 0.02 ^c^	2.21 ± 0.03 ^b^	2.37 ± 0.03 ^a^	100 ± 0.00
Crucian carp	1.80 ± 0.07	100.44 ± 0.44 ^a^	1.85 ± 0.05	1.24 ± 0.00 ^a^	2.33 ± 0.00 ^a^	1.96 ± 0.01 ^b^	100 ± 0.00
*p* value	1	<0.001	0.710	<0.001	<0.001	<0.001	

Different lowercase superscript letters within the same column indicate significant differences (*n* = 3). ICF, initial condition factor; WGR, weight gain rate; FCF, final condition factor (g/cm^3^); SGR, specific growth rate (%/d); FR, feeding rate (%); FCR, feed conversion ratio; SR, survival rate.

**Table 3:** Intestinal digestive enzyme activities in different ornamental fish species.

Items	Lipase (U/mg Prot)	Amylase (U/mg Prot)	Trypsin (U/mg Prot)
Sailfin molly	1.78 ± 0.15 ^b^	3.62 ± 0.22 ^b^	1.98 ± 0.14 ^b^
Goldfish	5.01 ± 0.04 ^a^	8.17 ± 0.23 ^a^	5.29 ± 0.18 ^a^
Red swordtail	5.05 ± 0.64 ^a^	8.38 ± 0.30 ^a^	5.44 ± 0.42 ^a^
Mickey Mouse platy	1.93 ± 0.33 ^b^	3.90 ± 0.25 ^b^	2.27 ± 0.16 ^b^
Golden crucian carp	1.79 ± 0.24 ^b^	3.68 ± 0.19 ^b^	2.11 ± 0.05 ^b^
Platinum mini parrot cichlid	2.07 ± 0.12 ^b^	4.28 ± 0.14 ^b^	2.51 ± 0.22 ^b^
Sapphire mini parrot cichlid	1.77 ± 0.22 ^b^	3.75 ± 0.37 ^b^	2.18 ± 0.30 ^b^
Crucian carp	4.96 ± 0.47 ^a^	8.29 ± 0.24 ^a^	5.37 ± 0.13 ^a^
*p* value	<0.001	<0.001	<0.001

Different superscript lowercase letters in the same column indicate significant differences (*n* = 3).

**Table 4:** Morphological indices of intestinal tissue of different ornamental fish species.

Items	Muscular Thickness (μm)	Villi Length (μm)	Villi Width (μm)	Goblet Cell Area (μm^2^)	Intestinal Wall Thickness (μm)	Mucosal Thickness (μm)
Sailfin molly	16.87 ± 2.03 ^e^	152.33 ± 8.65 ^e^	68.37 ± 3.33 ^c^	8484.7 ± 193.4 ^b^	173.89 ± 28.12 ^d^	165.48 ± 20.47 ^de^
Goldfish	117.73 ± 3.26 ^a^	245.07 ± 9.32 ^cd^	97.70 ± 3.98 ^a^	2814.2 ± 149.5 ^d^	378.89 ± 25.67 ^b^	313.34 ± 43.21 ^bc^
Red swordtail	36.63 ± 3.08 ^c^	553.23 ± 40.51 ^a^	87.43 ± 5.70 ^ab^	9449.0 ± 455.1 ^a^	607.55 ± 12.83 ^a^	594.98 ± 27.67 ^a^
Mickey Mouse platy	17.30 ± 1.37 ^e^	272.20 ± 17.42 ^bcd^	80.07 ± 2.13 ^bc^	8558.0 ± 367.5 ^ab^	354.32 ± 27.16 ^b^	295.26 ± 19.75 ^c^
Golden crucian carp	22.27 ± 2.76 ^de^	149.30 ± 10.96 ^e^	76.30 ± 5.25 ^bc^	5497.8 ± 327.9 ^c^	161.14 ± 21.22 ^d^	154.43 ± 23.12 ^e^
Platinum mini parrot cichlid	26.07 ± 1.88 ^d^	297.50 ± 18.64 ^bc^	95.60 ± 6.01 ^a^	2931.5 ± 120.9 ^d^	354.01 ± 39.36 ^b^	309.55 ± 29.26 ^bc^
Sapphire mini parrot cichlid	20.90 ± 2.11 ^de^	209.33 ± 23.17 ^de^	78.97 ± 2.26 ^bc^	3407.4 ± 110.2 ^d^	230.04 ± 26.17 ^c^	224.59 ± 36.86 ^d^
Crucian carp	59.20 ± 1.65 ^b^	321.60 ± 24.52 ^b^	97.93 ± 6.00 ^a^	1718.9 ± 85.0 ^e^	395.11 ± 25.19 ^b^	389.56 ± 32.56 ^b^
*p* value	<0.001	0.004	<0.001	<0.001	<0.001	<0.001

Different superscript lowercase letters in the same column indicate significant differences (*n* = 6).

**Table 5:** Comparison of hepatocyte perimeter and area among different ornamental fish species.

Items	Cell Perimeter (C/μm)	Cell Area (S/μm^2^)
Sailfin molly	46.28 ± 4.05 ^ab^	149.47 ± 16.69 ^bc^
Goldfish	45.80 ± 3.45 ^ab^	147.39 ± 20.66 ^bc^
Red swordtail	58.96 ± 5.61 ^a^	244.51 ± 26.92 ^a^
Mickey Mouse platy	32.71 ± 3.18 ^b^	74.78 ± 14.73 ^d^
Golden crucian carp	47.42 ± 4.42 ^ab^	159.65 ± 16.55 ^bc^
Platinum mini parrot cichlid	42.17 ± 7.94 ^ab^	128.38 ± 22.30 ^cd^
Sapphire mini parrot cichlid	53.71 ± 4.89 ^a^	190.55 ± 20.06 ^ab^
Crucian carp	44.1 ± 3.75 ^ab^	136.59 ± 23.92 ^cd^
*p* value	0.022	0.015

Different superscript lowercase letters in the same column indicate significant differences (*n* = 6).

## Data Availability

The raw data supporting the findings of this study are available from the corresponding author on reasonable request.

## Supplementary Materials

The following supporting information can be downloaded at: https://www.mdpi.com/article/10.3390/biology15131043/s1, Figure S1: Diagram of eight ornamental fish; Table S1: Some original data for WGR of eight rnamental fish.
