# Supplementary material for: Comparison of Morphological Characteristics, Histological Tissue Structures, and Intestinal Function Among Eight Ornamental Fish Species Under Identical Aquaculture Conditions
Source: Biology (Basel). 2026 Jun 30;15(13):1043. doi: 10.3390/biology15131043 (PMC13359742; doi:10.3390/biology15131043)
Supplement: Supplementary file 1 [file biology-15-01043-s001.zip › biology-4360753-supplementary.pdf]

Supplementary Materials

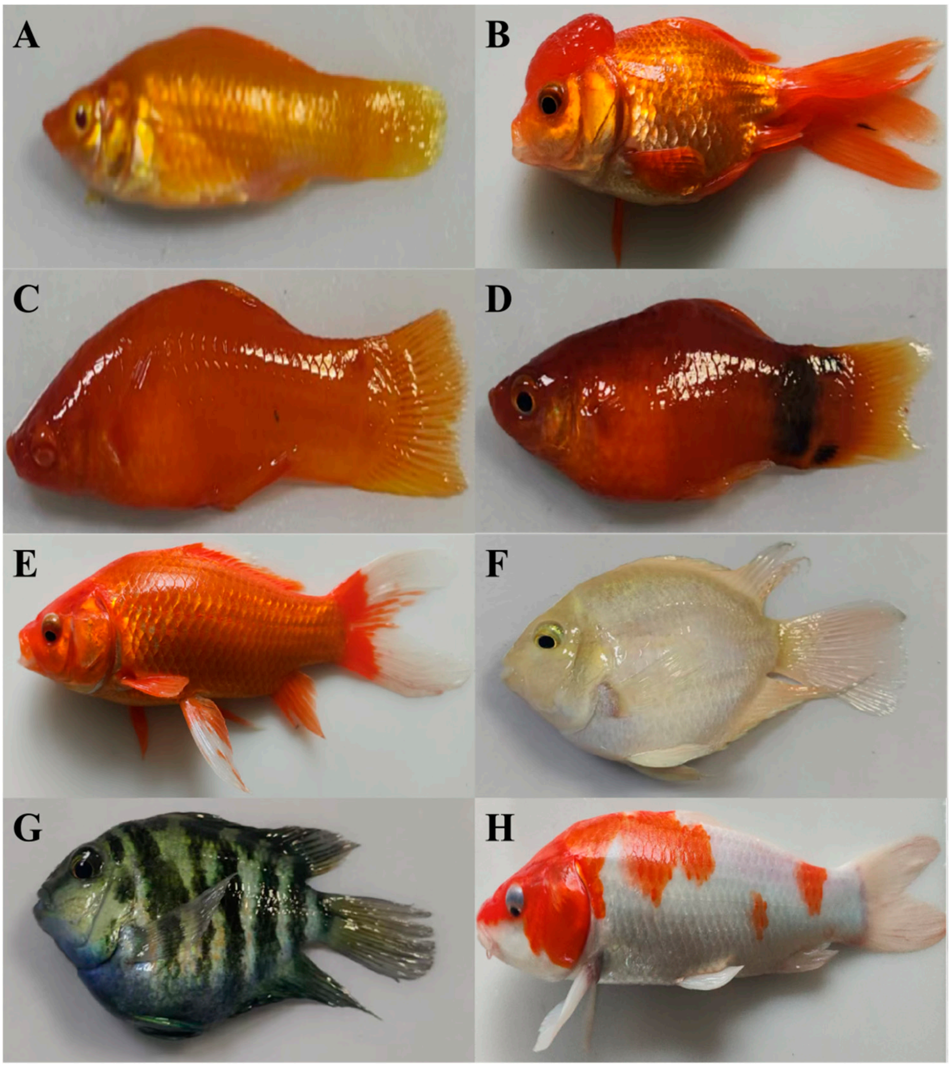

Figure S1. Diagram of eight ornamental fish. Sailfin molly (A), goldfish (B), red swordtail (C), Mickey Mouse platy (D), golden crucian carp (E), platinum mini parrot cichlid (F), sapphire mini parrot cichlid (G), and crucian carp (H).(All photographs were photographed by Yan Chen, Hainan Key Laboratory for Conservation and Utilization of Tropical Marine Fishery Resources. All images are original and have not been published elsewhere).

Table S1 Some original data for WGR of eight rnamental fish

Fish	IBW (g)	FBW (g)	WGR(%)
Sailfin molly	2.95	4.95	67.80
	3.00	5.25	75.00
	3.00	5.10	70.00
Goldfish	26.80	53.80	100.75
	27.50	55.00	100.00
	27.50	54.90	99.64
Red swordtail	8.90	15.00	68.54
	9.40	15.90	69.15
	9.40	15.90	69.15
Mickey Mouse platy	.70	1.20	71.43
	1.10	1.95	77.27
	1.00	1.85	85.00
Golden crucian carp	33.00	66.00	100.00
	33.80	67.60	100.00
	33.90	67.80	100.00
Platinum mini parrot cichlid	7.10	12.20	71.83
	7.50	12.80	70.67
	7.50	12.70	69.33
Sapphire mini parrot cichlid	7.80	13.50	73.08
	8.40	14.10	67.86
	8.50	14.10	65.88
Crucian carp	29.80	59.60	100.00
	30.50	61.00	100.00
	30.40	61.20	101.32
